# Case report: rare case of infiltration of small lymphocytic B-cell lymphoma in the thyroid gland of female patient with B-cell chronic lymphocytic leukemia (CLL-B/SLL-B)

**DOI:** 10.1186/1756-6614-6-1

**Published:** 2013-01-02

**Authors:** Elżbieta Andrysiak-Mamos, Rafał Becht, Elżbieta Sowińska-Przepiera, Jakub Pobłocki, Justyna Syrenicz, Barbara Zdziarska, Katarzyna Karpińska-Kaczmarczyk, Anhelli Syrenicz

**Affiliations:** 1 Department of Endocrinology, Metabolic Diseases and Internal Diseases, Pomeranian Medical University, ul. Unii Lubelskiej 1, 70-252, Szczecin, Poland; 2 Department of Hematology, Pomeranian Medical University, Szczecin, Poland; 3 Department of Pathology, Pomeranian Medical University, Szczecin, Poland

**Keywords:** Chronic lymphocytic leukemia, CLL, Cytometry, Lymphoma, Thyroid

## Abstract

The article presents a case of 57-year-old woman with the infiltration of rare small lymphocytic B cell lymphoma in the thyroid gland. Initially, the patient was followed-up due to chronic lymphocytic B-cell leukemia diagnosed on the basis of histopathological examination of cervical lymph node. Eight months later, general symptoms occurred along with lymphocytosis and exacerbation of lesions in lymph nodes, and therefore, chemotherapy was started according to COP regimen. After four chemotherapy cycles, further progression of the disease was observed during chemotherapy. Computed tomography (CT) performed at that time showed generalized lymphadenopathy and the presence of an irregular area in left thyroid lobe. On palpation, the thyroid was asymmetrical, with enlarged left lobe and palpable lymph node packages on the left side of the neck. The levels of thyroid hormones and anti-thyroid antibodies were normal. Ultrasound examination of the thyroid gland showed non-homogeneous hypoechogenic structure of the left lobe and complete focal remodeling. Cytological examination of left-lobe lesion obtained during fine needle aspiration biopsy showed multiple small lymphoid cells, suggestive of small lymphocytic lymphoma. To confirm this diagnosis, flow cytometry of the biopsy material sampled from the left lobe was performed showing B cellimmunophenotype: CD19+/CD20+/CD22 dim/FMC-7, CD23+/CD5+, sCD79b-+, CD38-, CD10-, kappa and lambda-/weak reaction. The results of flow cytometry of the thyroid bioptate and blood were nearly identical, confirming leukemic nature of the infiltration in left thyroid lobe. Cytogenetic findings included the presence of 17p deletion (TP53 gene). The patient received immunochemotherapy with alemtuzumab. The progression of the disease occurred in the sixth week of therapy. The treatment was discontinued after 8 weeks due to worsening of patient’s general status. The patient died 15 months after the diagnosis.

## Background

Lymphomas account for approximately 2% of all malignancies of the thyroid gland, while the lymphomas with primary location in the thyroid gland constitute about 2.5% of all lymphomas [1,2]. Normal thyroid gland does not contain lymphatic tissue; its presence is characteristic for pathological conditions, mainly as a result of the transformation of autoimmune thyroiditis. Hashimoto’s thyroiditis most often coexists with MALT (mucosa-associated lymphoid tissue), which represents approximately 23% of thyroid lymphomas [2]. The most frequent histologic type of thyroid lymphomas is diffuse large B-cell lymphoma (DLCL), which accounts for about 50% of all cases. Small lymphocytic B-cell lymphoma (SLL-B) is an extremely rare type of thyroid lymphoma (about 4% of cases). According to the classification presented by World Health Organization in 2008, B-cell chronic lymphocytic leukemia (CLL-B) is a leukemic form of SLL-B [3]. Pathological proliferation of B-cells occurs in patients with CLL-B; these cells accumulate as a result of impaired apoptosis, infiltrating bone marrow, lymph nodes, spleen and – in very rare cases – other organs [4,5]. This type of leukemia develops in patients between 65 and 70 years of age, more frequently in males, and it may remain asymptomatic for extended time. General symptoms, such as excessive sweating, weight loss, fever, and recurrent infections, including opportunistic infections, may occur at the beginning. Clinical picture includes lymphadenopathy and enlargement of the spleen and the liver. Peripheral blood cell count is characterized by leukocytic lymphocytosis; while, thrombocytopenia and anemia, including autoimmune hemolytic anemia, are less frequent [6]. Therapy depends on clinical stage of the disease and the presence of general symptoms. Apart from chemotherapy, immunochemotherapy with anti-CD20 and anti-CD52 monoclonal antibodies is used in the treatment of CLL-B.

## Case presentation

In December 2011, a 57-year-old patient was referred to Endocrinology Outpatient Clinic because of focal lesion found on CT, which was located in left thyroid lobe. The patient was monitored since March 2011 due to CLL-B. The diagnosis was based on clinical manifestation and histopathological examination of cervical lymph node. In January 2011, bilateral enlargement of cervical and axillary lymph nodes was found, without coexistent symptoms of systemic infection. Histopathological and immunohistochemistry findings in the specimen sampled from cervical lymph node led to the diagnosis of small lymphocytic B-cell lymphoma (SLL-B / CLL CD20(+), CD5(−), CD43(+), CD(23+), MIB-1 (+) [10% of cells]). The patient remained under the care of the Department of Hematology at Pomeranian Medical University. Treatment was not started, because the patient had no general symptoms and the disease was classified as stage I in Rai staging system (lymphocytosis and lymphadenopathy) as well as stage A in Binet classification (fewer than three areas of lymphoid involvement) [7]. Six months after the diagnosis, general symptoms occurred (excessive sweating, subfebrile temperature) together with the progression of changes in lymph nodes and lymphocytosis. Chemotherapy according to COP regimen (cyclophosphamide, vincristine, and prednisone) was started, with each cycle lasting 21 days. After completion of 4 treatment cycles, general symptoms exacerbated and progression of changes in the lymph nodes was found. In December 2011 (10 months after the diagnosis), CT revealed generalized lymphadenopathy (the enlargement of cervical, axillary, mediastinal, retroperitoneal, and inguinal lymph nodes) as well as the presence of an irregular, 22-mm long area with calcifications located in left thyroid lobe, which prompted endocrine diagnostic investigations.

**Figure 1:**
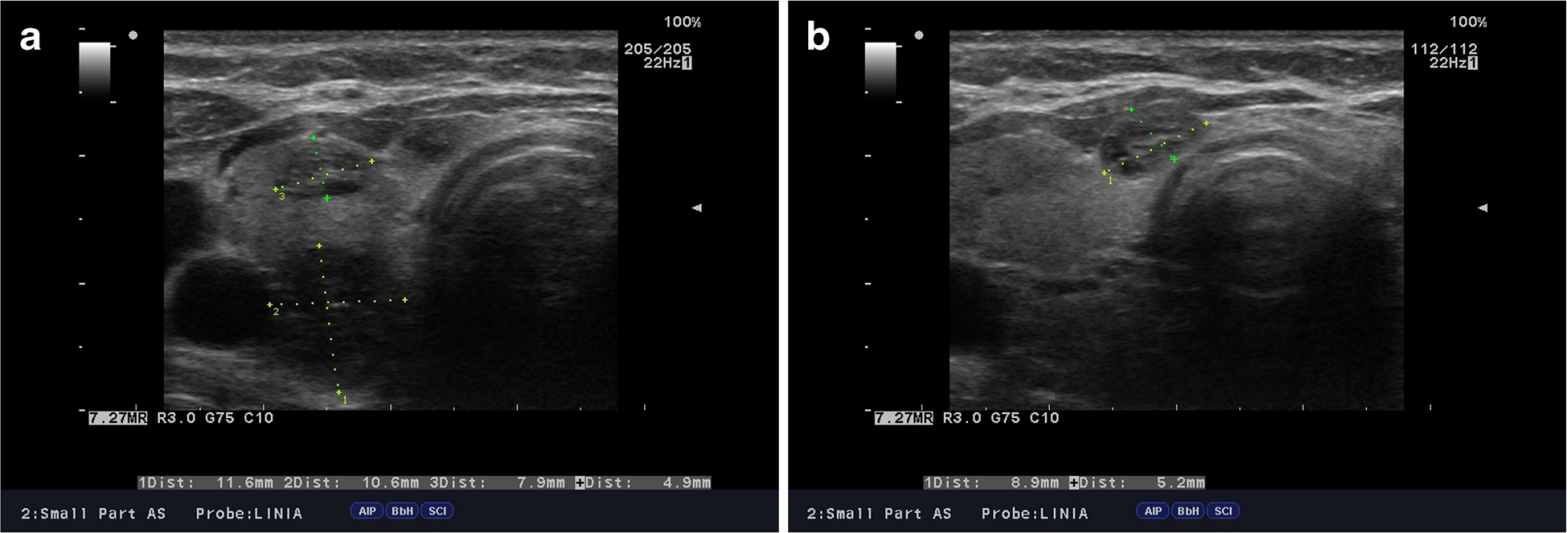
(a,b) Ultrasound image of right thyroid lobe with the features of nodular remodeling in 57-year-old female with SLL-B/CLL-B.

**Figure 2:**
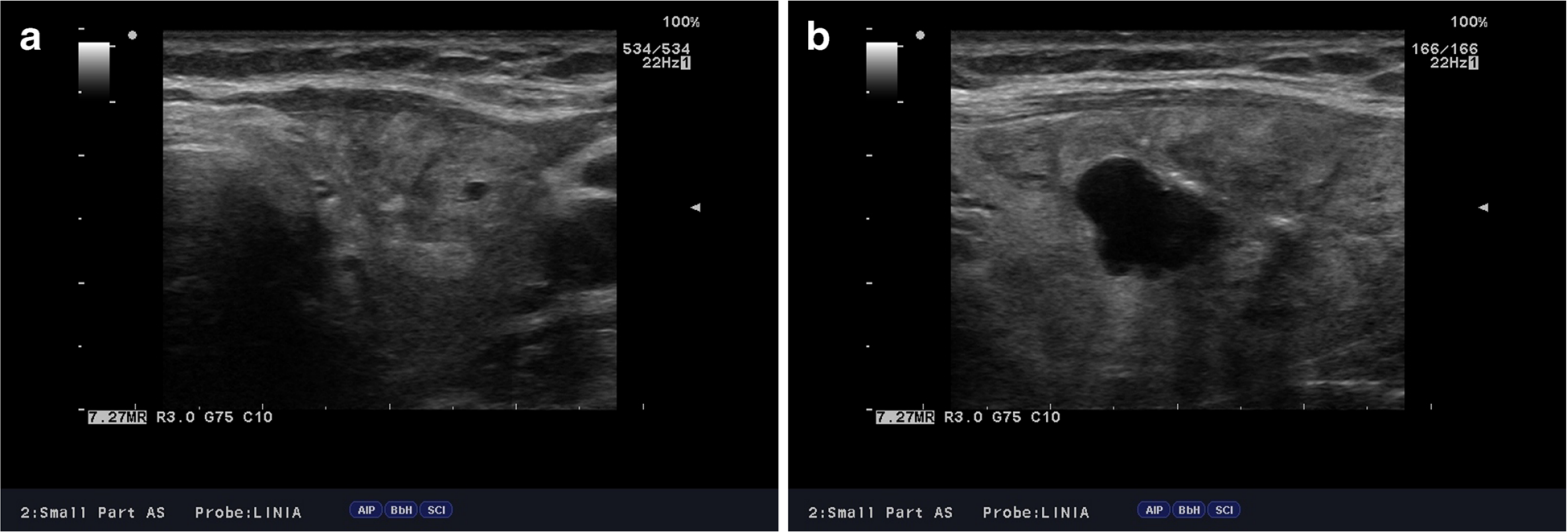
(a,b) Ultrasound image of left thyroid lobe with the infiltration of small lymphocytic B-cell lymphoma in 57-year-old female with SLL-B/CLL-B.

**Figure 3:**
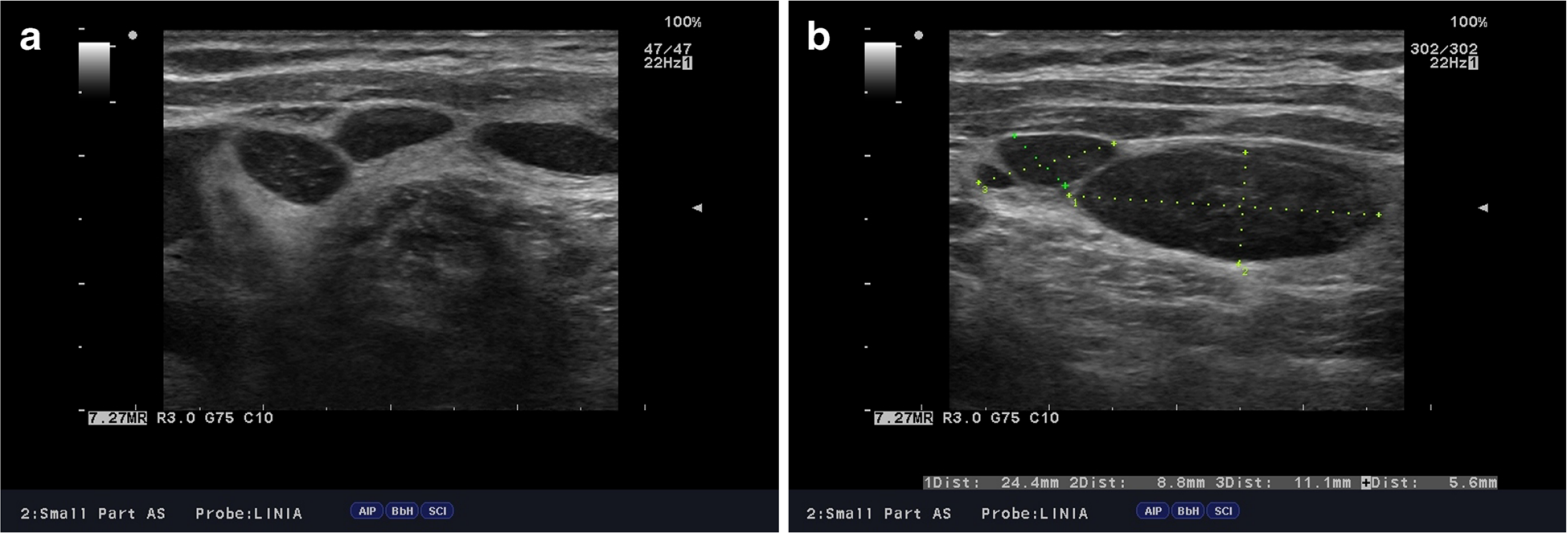
(a,b) Ultrasound image of cervical lymph node packages located along the sternocleidomastoid muscle in 57-year-old female with SLL-B/CLL-B.

**Figure 4:**
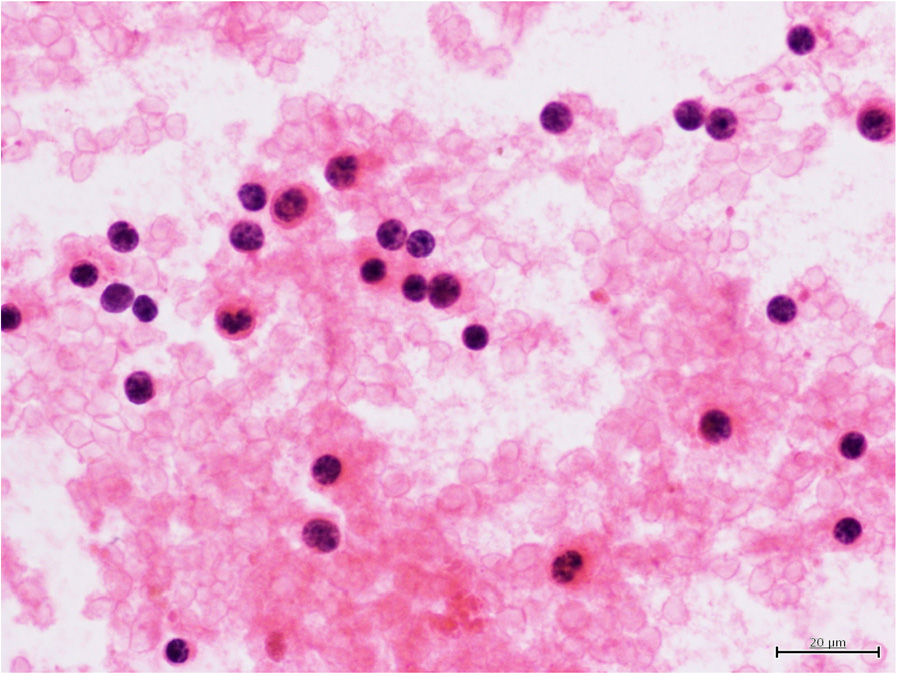
**Cytological smear composed of cells somewhat larger than small lymphocytes.** The nuclei appear spherical, the chromatin is coarsely granular and nuclei have regular contour. The nucleoli are not prominent. Single hemosiderophages are visible. The smears were fixed in alcohol and stained with hematoxylin-eosin (×400).

**Figure 5:**
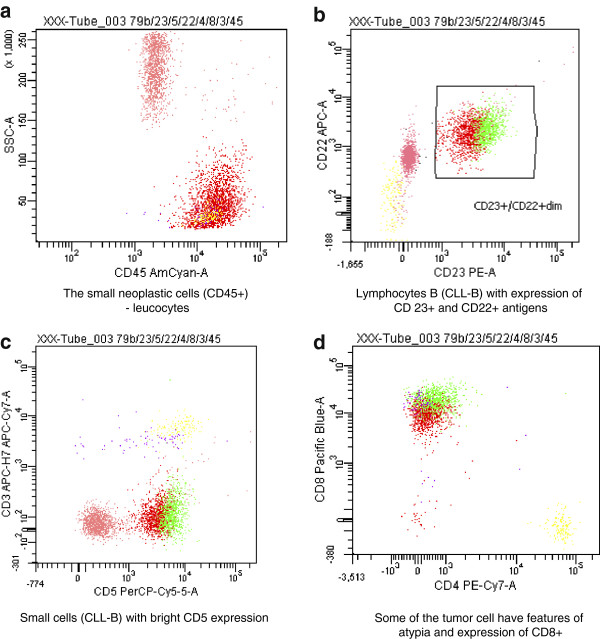
**(a-d) Flow cytometry interpretation.** The neoplastic cells display the typical antigenic features of CLL including small cell size and bright CD23 and CD5 expression. Some of the tumor cells have features of atypia and express CD8; these cells are considered to be more aggressive with azurophilic granules within cytoplasm. Yellow: CD3+/CD4-/CD8-; dark-blue: CD3+; red: CD23+/CD22 + dim; green: CD79b + dim.

On palpation, the thyroid was asymmetrical, with enlarged left lobe and without palpable nodules or audible thyroid bruit. Lymph node packages with the diameter of 5 cm were palpable on the lateral surface of the neck, particularly on its left side. Hormone tests revealed normal levels of free thyroid hormones and thyrotropin as well as normal levels of anti-thyroid peroxidase antibodies (anti-TPO), antithyroglobulin antibodies (anti-TG), anti-TSH receptor antibodies (TRAK), and normal thyroglobulin (TG) levels (Table 1). Ultrasound examination demonstrated asymmetry of the thyroid gland: left lobe was enlarged (59 × 23 × 21 mm), while the right lobe was normal in size (44 × 11 × 15 mm). The echostructure of the right lobe was heterogeneous with features of focal remodeling. Three focal lesions were found with the size of 9 × 5 mm, 8 × 5 mm and 12 × 11 mm (Figure 1). Left lobe had heterogeneous, hypoechogenic structure and was focally remodeled in its entirety. The largest focal lesion found in the left lobe was 35 × 23 mm large, was poorly separated, being solid–liquid in nature, and had heterogeneous, hypoechogenic structure with calcifications (Figure 2). Power Doppler ultrasound revealed vascularization of mixed type. Additionally, two more focal lesions were found in the left lobe: they were solid–liquid changes measuring 11 × 5 mm and 9 × 6 mm. Multiple packages of deeply hypoechogenic lymph nodes without visualized vascular hila and with pathologic vascularization were seen along the sternocleidomastoid muscles in Power Doppler ultrasound (Figure 3). Further diagnostic procedures included fine needle aspiration biopsy of the dominant lesion in left thyroid lobe, which showed multiple small lymphoid cells and isolated hemosiderinophages among blood constituents. Cytological picture of the lesion located in the left lobe suggested the infiltration of small lymphocytic lymphoma (Figure 4). The lesions found in the right thyroid lobe were described as benign. To confirm the diagnosis, flow cytometry of the biopsy material sampled from the focal lesion in left lobe was performed (Pathomorphology Unit at the Faculty of Medicine, Pomeranian Medical University in Szczecin) (Table 2). It was concluded that atypical cells from the sampled material have B-CLL immunophenotype: CD19+/CD20+/CD22dim/FMC-7,CD23+/CD5+,sCD79b-+,CD38-,CD10-, kappa and lambda - weak reaction (Figure 5). The results of flow cytometry of the biopsy materials sampled from the thyroid gland and blood were nearly identical. This fact confirmed the leukemic nature of the infiltration in left thyroid lobe.

**Table 1:** Hormonal parameters of female patient with SLL-B/CLL-B (at baseline and following 6-month follow-up)

**Parameter**	**Baseline**	**Follow-up**	**Reference range**
TSH [mIU/ml]	1.70	1.4	0.27-4.2
fT4 [ng/dl]	1.73	1.49	0.93-1.7
fT3[pg/ml]	3.36	3.12	1.8-4.6
Anti-TPO [IU/ml]	5.7	4.10	0-34
Anti-TG [IU/ml]	12.9	9.17	0-115
LDH [U/L]	713	868	135-214
B_2_ microglobulin [mg/L]	6.23	8.16	0.2-2.0

**Table 2:** Results of flow cytometry of fine needle biopsy specimen from the left thyroid lobe lesion

**Type of cell**	**Fraction [%]**
Mature lymphocytes CD45++ (CD3 83.3%, CD4 69%, CD8 7.9%, CD5 90%)	2%
Neutrophils (16+/10-+/11c+/11b+/CD33-)	36%
Cellular conglomerates, debris, necrotic material (?)	25%
Atypical cells (larger, with large granules; some of them undergo apoptosis despite retained antigen expression)	37%
Immunophenotype of atypical cells	CD20 – 100%, CD23 – 98.6%, CD5 – 99.4%, CD79b – 32.5%, CD22 – 41.8%, CD8 – 81.2%, CD56 – 91.4%, CD16 – 27.3%, CD71 – 93.3%, CD19 – 60.7%, CD13 – 35.2%, FMC-7 31.3%. Other antigens: CD3, CD4, CD38, CD11b, CD11c, CD10, kappa, and lambda were negative.

Due to the progression of the disease during chemotherapy, the diagnosis was re-verified, and suspicion was raised of a possible transformation of the disease into more malignant lymphoma. However, repeated histological examination of the lymph node, additional immunohistochemical examinations, and imaging examinations confirmed the diagnosis of CLL-B, also known as SLL-B. The patient also underwent cytogenetic examinations, which demonstrated the presence of 17p deletion (TP53 gene). The patient was qualified for immunochemotherapy with alemtuzumab (anti-CD52 monoclonal antibody). The treatment was started in January 2012 at the Department of Hematology, Pomeranian Medical University in Szczecin, and it was continued at the Chemotherapy Day Unit of the Department of Hematology. In the first weeks of treatment, the size of lymph nodes was reduced below 50% of baseline values and general symptoms resolved. Nevertheless, it should be emphasized that the infiltration found in the left lobe did not diminish in size, but instead slightly enlarged. Another progression of the disease occurred in the sixth week of treatment. After eight weeks, therapy was discontinued due to further progression of the disease and deterioration in patient’s general status. Despite anti-viral prophylactic measures, the patient developed generalized form of zoster. In the course of further treatment, the patient was qualified for palliative radiotherapy of lymph nodes. During radiotherapy, the patient suffered from progressive paresis of the limbs as the spinal cord became affected by the underlying disease. The patient died 15 months after diagnosis due to the progression of the disease, despite chemotherapy and immunochemotherapy with alemtuzumab.

Written consent was obtained from the patient's family for publication of this report and any accompanying images.

## Discussion

The case of SLL-B presented by our team belongs to the least common subtypes of thyroid lymphoma. It is often associated with the involvement of lymph nodes, bone marrow, spleen, liver and – extremely rarely – other organs. Our patient developed generalized lymphadenopathy and the infiltration of left thyroid lobe, without hepatosplenomegaly and with low-grade leukocytosis. Similar location of changes and asymptomatic onset of the disease was described by Shin et al. in another female patient [8]. It is believed that the history of chronic autoimmune Hashimoto’s thyroiditis predisposes to the occurrence of lymphoma lesions in the thyroid [9,10]. According to literature, 80% of thyroid lymphomas are preceded by long-lasting (up to 20-30-year long) Hashimoto’s thyroiditis [11]. This kind of thyroiditis particularly predisposes to the development of DLBCL lymphoma and MALT lymphoma, and it may often hinder the assessment of cytological findings and make the final diagnosis difficult [1]. Our patient did not present autoimmune changes in the thyroid gland or elevated levels of anti-thyroid antibodies and thyroid dysfunction at the time of CLL-B diagnosis.

There are four reports in literature on small lymphocytic B-cell lymphoma (SLL-B/CLL-B), coexisting with cancers or thyroiditis [8,12-14]. Two of the cases described in literature involved the coexistence of SLL-B/CLL-B and papillary cancer or papillary and medullary cancer of the thyroid gland [12,13]. In the remaining two cases described by Shin et al. [8] and Trümper et al. [14], the development of lymphoma of the thyroid gland was preceded by autoimmune thyroiditis with elevated levels of anti-thyroid peroxidase antibodies and anti-thyroglobulin antibodies [8,14]. Flow cytometry performed in these cases and in our patient showed similar expression of B-cell antibodies: CD20, CD19, CD5, and CD23. Although our patient did not present biochemical data indicative of autoimmune thyroiditis, ultrasound image of left thyroid lobe might have suggested such diagnosis. It should be emphasized that the diagnosis of lymphoma based on fine needle aspiration biopsy is not always possible; it may be especially difficult to establish its histologic subtype and, consequently, to decide about further treatment and prognosis. Core-needle biopsy, or intraoperative sampling of the specimens, is recommended [1,15]. Immunohistochemistry and flow cytometry are useful for the differentiation between Hashimoto’s thyroiditis, lymphoma, and poorly differentiated or anaplastic cancer of the thyroid. It is easier to arrive at the final diagnosis of focal thyroid lesions, if the patient already has the history of CLL-B, as it was the case in our patient. It may also happen that thyroid changes are diagnosed first and only later the diagnosis of SLL-B/CLL-B is established [8,12].

The treatment of primary lymphomas of the thyroid gland, as well as infiltration changes associated with CLL-B, remains controversial. The recommended strategy includes chemotherapy, radiotherapy, and in particular cases also surgery and immunochemotherapy with monoclonal antibodies. Our patient did not respond to chemotherapy and progression of the disease was observed during treatment. Because of genetic background of the disease (17p13 deletion TP53 gene), alemtuzumab (anti-CD52 monoclonal antibody) was started. Nearly 5% of patients show 17p13 deletion at the time of CLL-B diagnosis, but during the course of the disease 17p13 deletion is found in almost 50% of the patients. These genetic changes are associated with the resistance to standard treatment and they are an unfavorable prognostic factor [16,17]. The use of alemtuzumab in patients with CLL-B and 17p13 deletion caused positive response only in 40% patients [18]. This drug is also recommended in patients with CLL-B and other genetic disorders, e.g. 11q22 deletion (positive response in 27% patients) [19]. In our patient, alemtuzumab treatment did not result in the regression of thyroid lesions, despite good initial response to the treatment as was reflected in the reduced size of lymph nodes. Dynamic progression of the disease and 15-month long survival despite treatment are likely to be associated with genetic background of the disease, the presence of TP53 gene mutation – 17p deletion, which, as many authors emphasize, is an unfavorable prognostic factor.

## Conclusions

The occurrence of small lymphocytic B-cell lymphoma (SLL-B) in the thyroid gland associated with CLL-B may not be preceded by autoimmune thyroiditis (Hashimoto’s thyroiditis).

Thyroid lesions associated with SLL-B/CLL-B do not respond to treatment (chemotherapy and immunochemotherapy – with alemtuzumab).

The presence of 17p deletion is an unfavorable prognostic factor in patients with SLL-B/CLL-B.

## Competing interests

The authors declare that they have no competing interests.

## Authors’ contributions

EAM, ESP, and AS conceived of the study, participated in its design and coordination, and helped to draft the manuscript. RB, JP, JS, BZ, and KKK conceived of the study and helped to draft the manuscript. All authors read and approved the final manuscript.

## References

[B1] Sangalli G Serio G Zampatti C Lomuscio G Colombo L Fine needle aspiration cytology of primary lymphoma of the thyroid: a report of 17 cases Cytopathology 2001 12 257 263 10.1046/j.1365-2303.2001.00338.x 11488875

[B2] Thieblemont C Mayer A Dumontet C Barbier Y Callet-Bauchu E Felman P Berger F Ducottet X Martin C Salles G Orgiazzi J Coiffier B Primary thyroid lymphoma is a heterogeneous disease J Clin Endocrinol Metab 2002 87 105 111 10.1210/jc.87.1.105 11788631

[B3] Harris NL Jaffe ES Diebold J Flandrin G Muller-Hermelink HK Vardiman J Lister TA Bloomfield CD The World Health Organization classification of neoplastic diseases of the hematopoietic and lymphoid tissues. Report of the clinical advisory committee meeting, Airlie house, Virginia, November, 1997 Ann Oncol 1999 10 1419 1432 10.1023/A:1008375931236 10643532

[B4] Derringer GA Thompson LD Frommelt RA Bijwaard KE Heffess CS Abbondanzo SL Malignant lymphoma of the thyroid gland: a clinicopathologic study of 108 cases Am J Surg Pathol 2000 24 623 639 10.1097/00000478-200005000-00001 10800981

[B5] Kossev P Livolsi V Lymphoid lesions of the thyroid: review in light of the revised European-American lymphoma classification and upcoming World Health Organization classification Thyroid 1999 9 1273 1280 10.1089/thy.1999.9.1273 10646671

[B6] Graff-Baker A Roman SA Thomas DC Udelsman R Sosa JA Prognosis of primary thyroid lymphoma: demographic, clinical, and pathologic predictors of survival in 1,408 cases Surgery 2009 146 1105 1115 10.1016/j.surg.2009.09.020 19958938

[B7] Rai KR Sawitsky A Cronkite EP Chanana AD Levy RN Pasternack BS Clinical staging of chronic lymphocytic leukemia Blood 1975 46 219 234 27789434 10.1182/blood-2016-08-737650

[B8] Shin J Chute D Milas M Mitchell J Siperstein A Berber E A rare case of chronic lymphocytic leukemia/small lymphocytic lymphoma presenting in the thyroid gland Thyroid 2010 20 1019 1023 10.1089/thy.2010.0089 20718685

[B9] Holm LE Blomgren H Lowhagen T Cancer risks in patients with chronic lymphocytic thyroiditis N Engl J Med 1985 312 601 604 10.1056/NEJM198503073121001 3838363

[B10] Saxena A Alport EC Moshynska O Kanthan R Boctor MA Clonal B cell populations in a minority of patients with Hashimoto’s thyroiditis J Clin Pathol 2004 57 1258 1263 10.1136/jcp.2004.018416 15563664 PMC1770528

[B11] Pedersen RK Pedersen NT Primary non-Hodgkin’s lymphoma of the thyroid gland: a population based study Histopathology 1996 28 25 32 10.1046/j.1365-2559.1996.268311.x 8838117

[B12] Bocian A Kopczynski J Rieske P Piaskowski S Sluszniak J Kupnicka D Gozdz S Kowalska A Sygut J Simultaneous occurrence of medullary and papillary carcinomas of the thyroid gland with metastases of papillary carcinoma to the cervical lymph nodes and the coinciding small B-cell lymphocytic lymphoma of the lymph nodes-a case repor Pol J Pathol 2004 55 23 30 15619977

[B13] Reid-Nicholson M Moreira A Ramalingam P Cytologic features of mixed papillary carcinoma and chronic lymphocytic leukemia/small lymphocytic lymphoma of the thyroid gland Diagn Cytopathol 2008 36 813 817 10.1002/dc.20894 18831028

[B14] Trumper L Matthaei-Maurer DU Knauf W Moller P Centroblastic lymphoma of the thyroid supervening long-lasting chronic lymphocytic leukemia (B-CLL) demonstration of biclonality by immunohistochemical and gene rearrangement analysis Klin Wochenschr 1988 66 736 742 10.1007/BF01726417 3139912

[B15] Takashima S Sone S Horii A Honjho Y Yoshida J Subacute thyroiditis in Hashimoto’s thyroiditis which mimicked primary thyroid lymphoma J Clin Ultrasound 1997 25 279 281 10.1002/(SICI)1097-0096(199706)25:5<279::AID-JCU11>3.0.CO;2-E 9314112

[B16] Dohner H Fischer K Bentz M Hansen K Benner A Cabot G Diehl D Schlenk R Coy J Stilgenbauer S p53 gene deletion predicts for poor survival and non-response to therapy with purine analogs in chronic B-cell leukemias Blood 1995 85 1580 1589 7888675

[B17] Dohner H Stilgenbauer S Benner A Leupolt E Krober A Bullinger L Dohner K Bentz M Lichter P Genomic aberrations and survival in chronic lymphocytic leukemia N Engl J Med 2000 343 1910 1916 10.1056/NEJM200012283432602 11136261

[B18] Lozanski G Heerema NA Flinn IW Smith L Harbison J Webb J Moran M Lucas M Lin T Hackbarth ML Proffitt JH Lucas D Grever MR Byrd JC Alemtuzumab is an effective therapy for chronic lymphocytic leukemia with p53 mutations and deletions Blood 2004 103 3278 3281 10.1182/blood-2003-10-3729 14726385

[B19] Stilgenbauer S Dohner H Campath-1H-induced complete remission of chronic lymphocytic leukemia despite p53 gene mutation and resistance to chemotherapy N Engl J Med 2002 347 452 453 10.1056/NEJM200208083470619 12167696

